# New and innovative biomaterials, techniques and therapy concepts: Biologization in maxillofacial surgery, oral surgery and dentistry is in full swing. PRF, PRGF, PRP, blood plasma-stabilized augmentations, supplementation of micronutrients and vitamins – what opportunities do such “biological” approaches actually offer? We introduce them here.

**DOI:** 10.3205/iprs000166

**Published:** 2022-07-05

**Authors:** Ralf Smeets, Anders Henningsen, Oliver Zernial, Ewa Stürmer, Imke Fiedler, Sogand Schäfer, Martin Gosau, Robert Gaudin, Carolin Stolzer, Anna Reinelt, Sandra Fuest

**Affiliations:** 1 Department of Oral and Maxillofacial Surgery, University Medical Center Hamburg-Eppendorf, Hamburg, Germany; 2 Department of Oral and Maxillofacial Surgery, Division Regenerative Orofacial Medicine, University Medical Center Hamburg-Eppendorf, Hamburg, Germany; 3 Practice for Implantology, Maxillofacial and Aesthetic Facial Surgery, Kiel, Germany; 4 Department of Vascular Medicine, University Medical Center Hamburg-Eppendorf, Hamburg, Germany; 5 Institute of Osteology and Biomechanics, University Medical Center Hamburg-Eppendorf, Hamburg, Germany; 6 Department of Oral and Maxillofacial Surgery, Charité – Universitätsmedizin Berlin, Berlin, Germany

**Keywords:** dentistry, biomaterials, bone regeneration, tissue regeneration, silk, magnesium, 3D-printing, platelet-rich plasma, micronutrients

## Abstract

Biomaterials of natural origin have recently gained increasing attention in the field of dental implantology. The requirements for such materials, however, are very high. In addition to high clinical efficiency in tissue regeneration, wound healing should be demonstrably positively influenced. The translational division for regenerative orofacial medicine of the Clinic and Polyclinic for Oral and Maxillofacial Surgery of the University Medical Center Hamburg-Eppendorf (UKE) is examining this research topic by investigating which innovative treatment methods for the reconstruction of bone defects or for augmentative procedures can be applied in the future or are already being applied in the field of oral and maxillofacial surgery.

## Abstract (de)

Biomaterialien natürlichen Ursprungs im Bereich der dentalen Implantologie erhalten in den letzten Jahren vermehrt Aufmerksamkeit, jedoch sind die Anforderungen hoch. Neben einer hohen klinischen Effizienz bei der Geweberegeneration sollte die Wundheilung nachweislich positiv beeinflusst werden. Die translationale Sektion „Regenerative Orofaziale Medizin“ der Klinik und Poliklinik für Mund-, Kiefer- und Gesichtschirurgie der Uniklinik Hamburg-Eppendorf (UKE) beschäftigt sich mit diesem Thema und untersucht, welche innovativen Behandlungsmethoden zur Rekonstruktion von Knochendefekten oder bei augmentativen Verfahren im Bereich der Mund-, Kiefer- und Gesichtschirurgie zukünftig Anwendung finden oder bereits angewendet werden.

## 1 Introduction

From absorbable materials, such as silk or magnesium, to autologously derived platelet-rich plasma (PRP) from human blood, additive vitamin administration or 3D-printed implants: a variety of concepts are available to the treating physician or dentist, based on the current state of research, to facilitate the access to appropriate biomaterials for every implantological treatment option. The most important factors for successful treatment include properties, such as good biocompatibility, tissue integration, and cell occlusivity. Easy handling is also of great importance in a clinical setting in order to avoid unnecessary delays in treatment. But why is there a need for these innovative materials? How high is their success rate and for which areas of application in oral and maxillofacial surgery are they suitable? The following sections will provide an overview of current trends and techniques.

### 1.1 Current problems in hard and soft tissue management – clinical evidence

The range of functional materials in the field of dental implantology and surgery is extensive. Various materials, for example corrosion-resistant metallic materials such as titanium or non-metallic materials such as ceramics, are widely used for dental implants and osteosynthesis systems. Although these materials do not require any incremental optimization as they guarantee a high success rate for the patient due to high endurance and stability, they still carry risks which can lead to various disadvantages [1]. In addition to the risk of an allergic reaction, there is a long-term risk of developing mucositis or even peri-implantitis, which can lead to significant bone loss or even implant loss [2], [3].

Established biomaterials, absorbable as well as non-absorbable, are nowadays used as a new gold standard in the field of Guided Bone Regeneration/Guided Tissue Regeneration (GBR/GTR) or for orbital floor reconstruction [4]. Especially non-absorbable materials made of synthetic polymers, such as expanded polytetrafluoroethylene (ePTFE), are a foreign material for the human body which has to be removed in a second complex surgical procedure after the completion of surgical bone regeneration procedures. To circumvent these problems, absorbable materials have been successfully investigated and developed, for example, on the basis of collagen or of absorbable polymers, such as polylactides (PLA) [5]. However, up to now, some material-specific peculiarities of absorbable biomaterials are known, such as undesirable immune responses coupled with inhomogeneous tent stimulations, varying degradation times, increased costs and ethical problems regarding the extraction of animal derived materials that need to be considered [6]. Thus, an optimal application-specific biomaterial could not yet be determined. Although the range of possible applications is broad, implantation is often accompanied by an immune reaction since substitute biomaterials, such as fillers or barrier membranes, are recognized by the body as a foreign material. Biomaterial research has been advancing for more than 20 years to develop innovative materials that bypass the above-mentioned disadvantages and deliver improved biofunctional properties.

The challenge is to ensure that new material concepts guarantee cost-effective and process-automated production as well as ensuring the compatibility of implants while maintaining defect-filling functionality in the field of dental implantology.

## 2 Innovative concepts

### 2.1 Silk fibroin as a biomaterial

As an absorbable biomaterial, silk fibroin represents an interesting alternative to conventional materials such as collagen or absorbable biopolymers for soft tissue replacement. Based on the cocoons of the mulberry silkworm *Bombyx mori* (*B. mori*), these can be brought into an aqueous solution by a gentle dissolution process and then used as a starting material for further processing with a versatile and wide-ranging product portfolio. Especially, the customizable combination of their biological and mechanical properties is unique.

**Figure 1:**
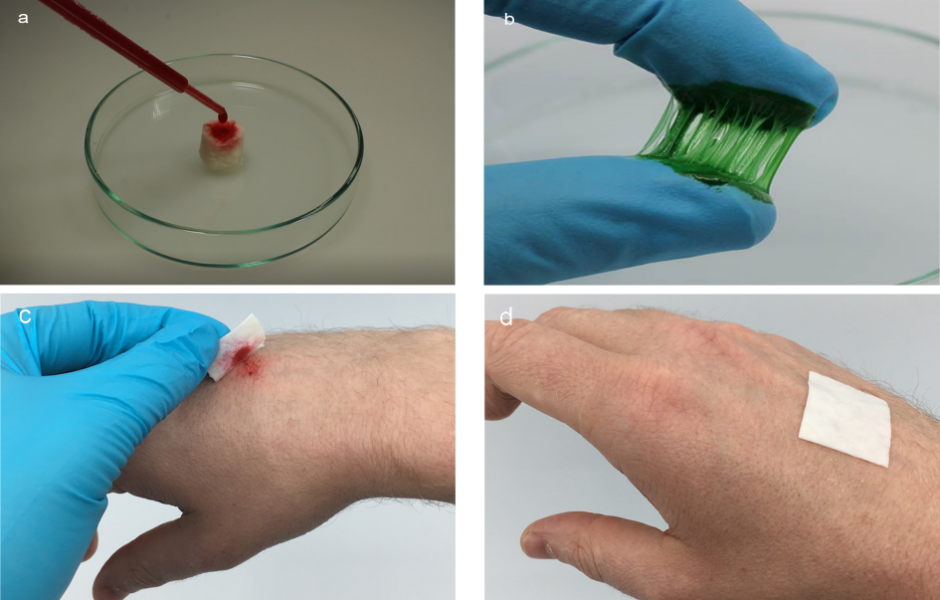
a: Macroporous sponge of silk fibroin as a hemostatic in coagulation-incompetent patients; b: absorbable silk-based wound adhesive; c-d: wound patches.

**Figure 2:**
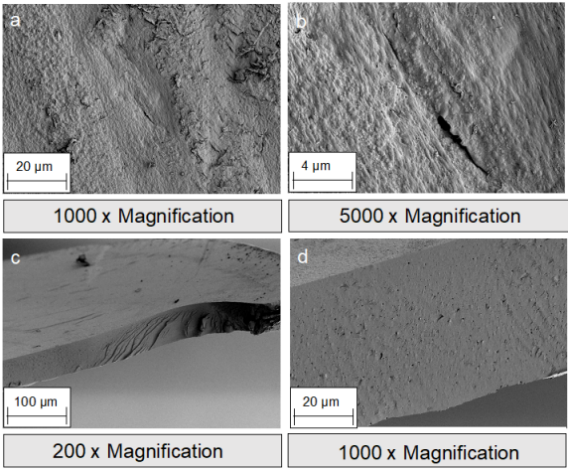
Scanning electron microscope (SEM) images of silk fibroin membranes: a-b: surface; c-d: cross section.

**Figure 3:**
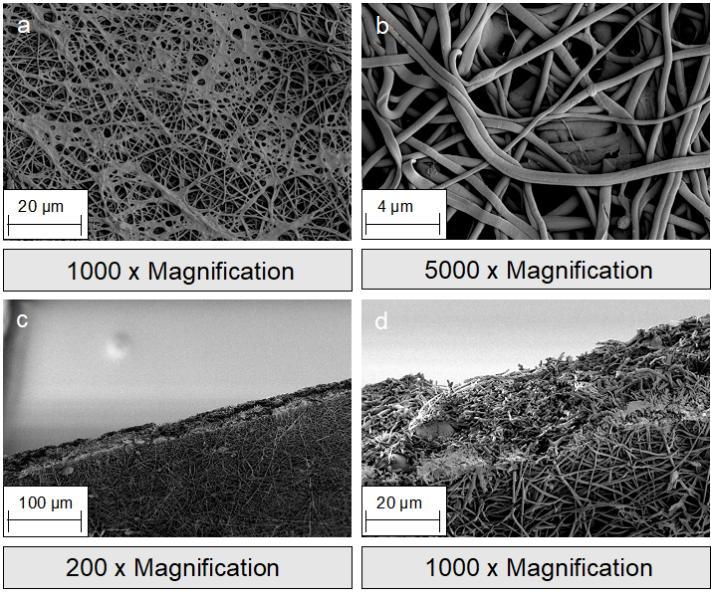
SEM images of silk fibroin nonwovens: a-b: surface; c-d: cross section.

**Figure 4:**
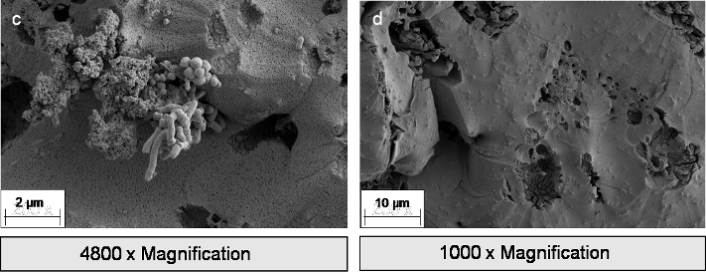
Multispecies biofilm on wound matrix. *Escherichia coli*, *Pseudomonas aeruginosa*, and *Candida albicans* surrounded by bacterial extrapolymeric substance (EPS). Magnification (SEM) 4,800x & 1,000x.

**Figure 5:**
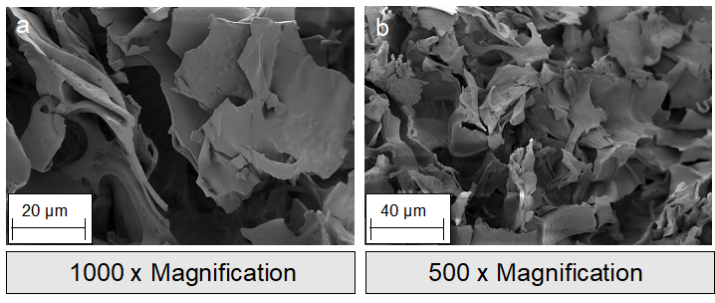
SEM images of silk fibroin sponges: surface

**Figure 6:**
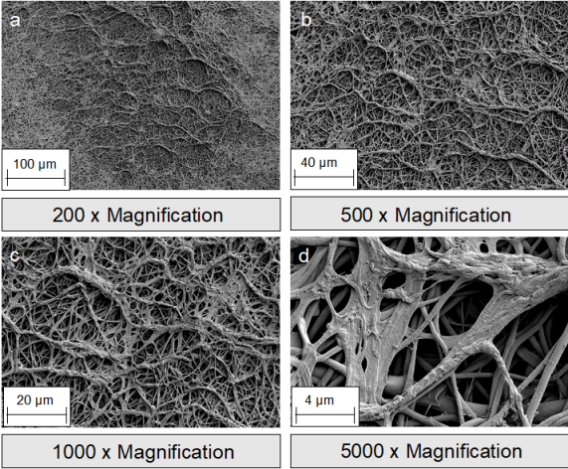
SEM images of antibacterial-loaded silk fibroin nonwovens

**Figure 7:**
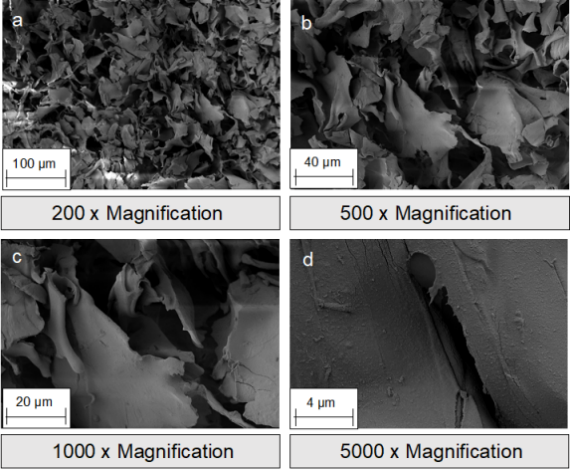
SEM images of antibacterial-loaded silk fibroin sponges

**Figure 8:**
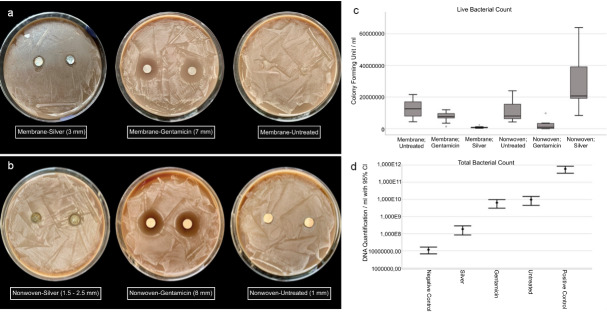
Antibacterial efficacy of functionalized fibroin membranes and nonwovens with silver: a-b: zone inhibition test; c: determination of live bacterial count; d: determination of total bacterial count.

The focus in oral and maxillofacial surgery includes macroporous 3D structures, such as tamponades and wound pads (absorbable patch, Figure 1c-d (Fig. 1)), novel wound adhesives (Figure 1b (Fig. 1)), and textile membranes for application in the field of GBR/GTR (Figure 1 (Fig. 1), Figure 2 (Fig. 2), Figure 3 (Fig. 3), Figure 4 (Fig. 4) and Figure 5 (Fig. 5)). Recent studies have investigated the antibacterial functionalization of silk structures (Figure 6 (Fig. 6) and Figure 7 (Fig. 7)): as a result, fibroin loaded with antibacterial agents, regardless of its structure, leads to a decrease in total and live bacterial cell count (Figure 4 (Fig. 4) and Figure 8 (Fig. 8)). The main focus currently lies on absorbable silk fibroin-based barrier membranes with and without an additionally embedded magnesium lattice structure. Here, the magnesium lattice has been shown not only to prevent membrane collapse into the defect but also to promote bone regeneration [7]. In current research series, the aforementioned silk structures show promising results for their future use in oral medicine.

However, biodegradable silk matrices can be useful in various other medical fields: Their resorption capacity and ability to absorb fluids (20 times their own weight, Figure 1a (Fig. 1)) make them an excellent bandage material for cutaneous wound healing. This way they can cover or fill defects, provide secretion control as well as a physiological wound environment due to their spongy form, and can even enhance the wound healing process through their antibacterial effect. This becomes especially apparent when this type of absorbable patches and sponges is used for controlled healing on split skin removal surfaces and flap lifting defects in oncological procedures in oral and maxillofacial surgery. Due to their absorbable character, such materials exhibit a high patient comfort as well as an economic advantage by avoiding frequent close-meshed bandage changes.

### 2.2 Magnesium as an absorbable material technology 

**Figure 9:**
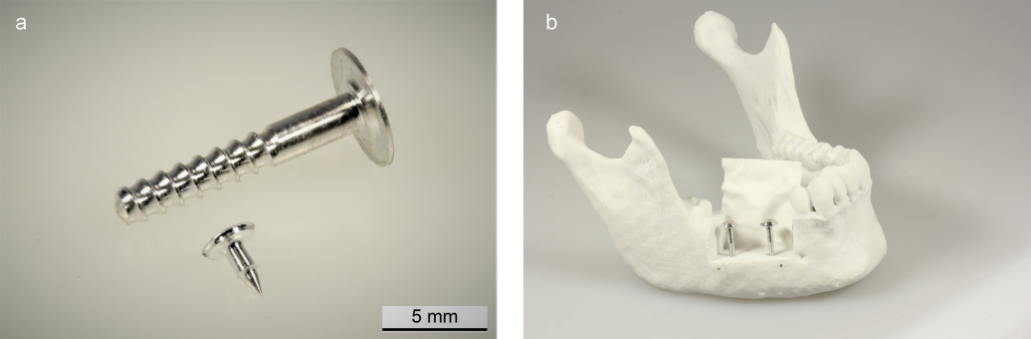
a: A metallic absorbable magnesium shielding screw and magnesium pin for membrane fixation for GBR/GTR; b: absorbable magnesium shielding screw for tentpole technique with silk membrane for defect coverage.

**Figure 10:**
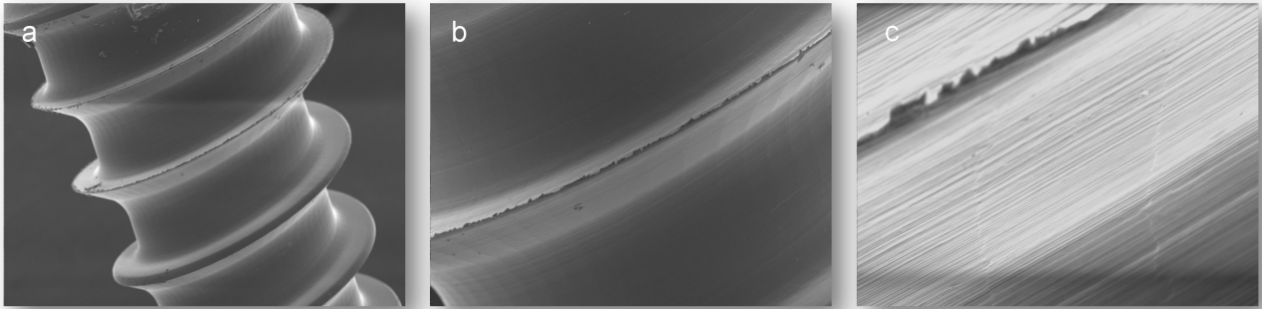
SEM images of absorbable magnesium osteosynthesis screws: a-c: representation of the thread of the screw with increasing magnification.

Another promising biodegradable material, which has higher mechanical strength and a lower likelihood of inflammatory reactions than comparable polymers, is magnesium and its alloys [8], [9]. Magnesium alloys represent the first metallic material class that has the potential to be used as a bone graft substitute in the human body due to their high biocompatibility, osseous strength, and continuous and controllable degradation behavior. The degradation behavior of magnesium is favored by the fact that it is an essential element for the human metabolism. Since there are no alternative absorbable implants with such an advantageous combination of properties in terms of mechanics and degradation behavior, so far, this technology is considered to have a high potential and is being investigated by many research groups as a revolutionary technology. In current studies, physicians are investigating magnesium lattice structures for the GBR/GTR technique as well as magnesium pins and screws for the so-called umbrella/tentpole technique (see Figure 9 (Fig. 9)). In the field of traumatology and trauma surgery, absorbable magnesium implants have already been used successfully for fracture treatment (Figure 10 (Fig. 10)) [10], [11], [12].

### 2.3 Additive manufacturing processes 

**Figure 11:**
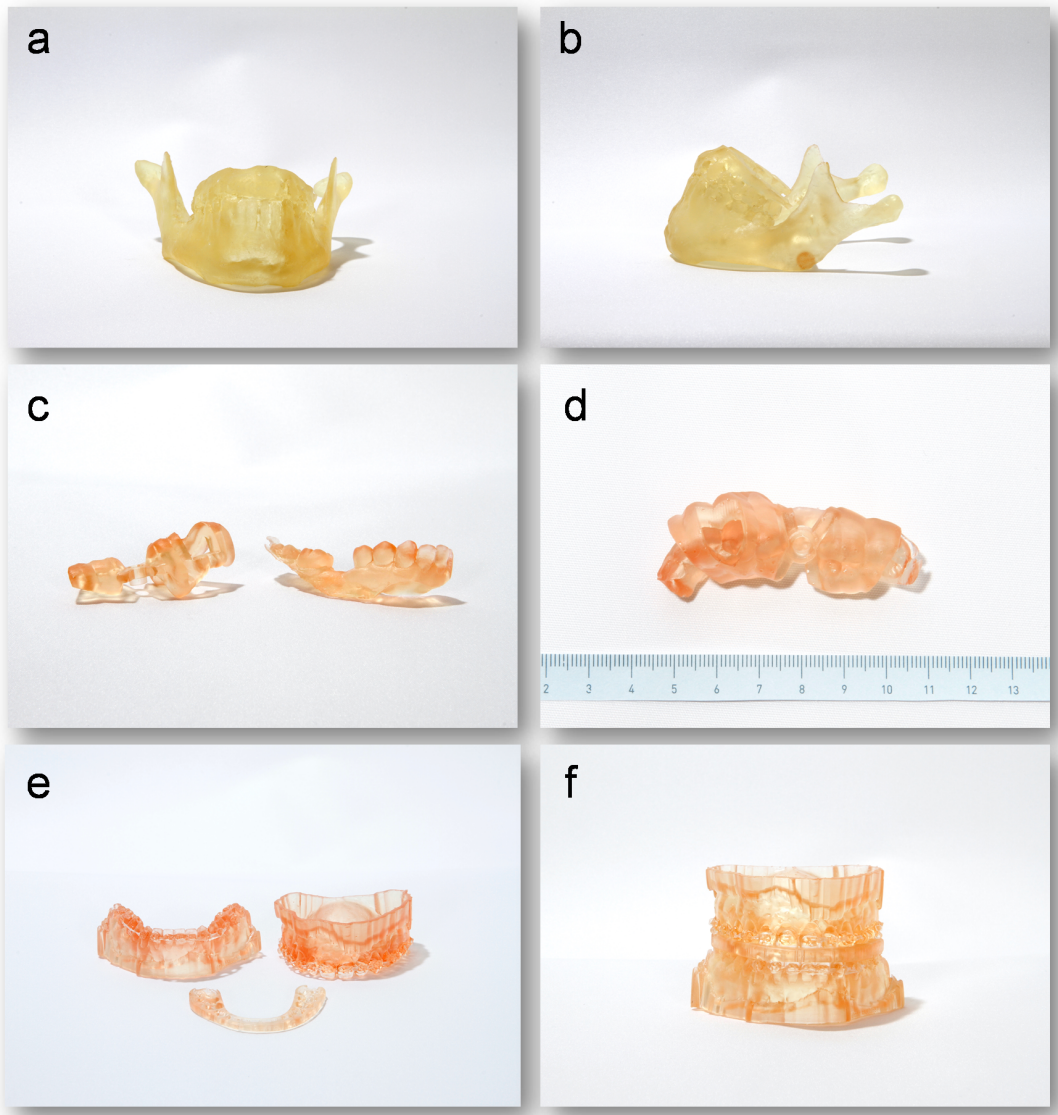
a-b: patient-specific models for preoperative planning; c-d: drill templates for implantation; e-f: splints for dysgnathia surgery.

**Figure 12:**
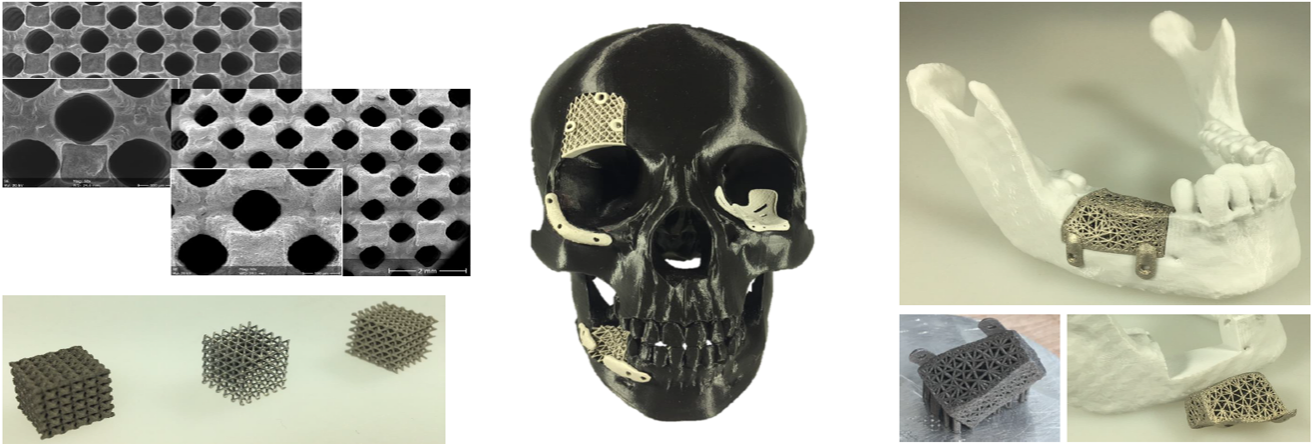
3D printing of magnesium implants using selective-laser-melting (SLM)

High-resolution and accurate imaging techniques are indispensable in everyday clinical practice: on the one hand, they permit an accurate diagnosis while on the other hand 3D data can be used for surgery to plan and perform complex operations as well as to manufacture individual 3D-printed implants [13], [14]. As of late, additive processing technologies such as 3D-printing or bioprinting can also be used [13], [14]. Currently, patient-specific models for preoperative planning (Figure 11a-b (Fig. 11)), drill guides for implant placement (Figure 11c-d (Fig. 11)) and splints for dysgnathia surgery (Figure 11e-f (Fig. 11)) are 3D printed for research purposes in our clinic. There is still a high need for research in this area before it can become part of an accepted clinical routine. However, innovative approaches that support tissue regeneration with 3D-printed implants or use human cells to generate structures that transform into organs over time show high potential. This is why the translational division for regenerative orofacial medicine of the UKE currently focuses on this research area amongst other things. Additionally, a separate research project is presently concentrating on 3D-printed absorbable magnesium implants (including individual skull implants (IPS), absorbable orbital floor meshes and individual reconstruction plates; Figure 12 (Fig. 12)).

### 2.4 Abutment and implant surfaces / antibacterial coating 

**Figure 13:**
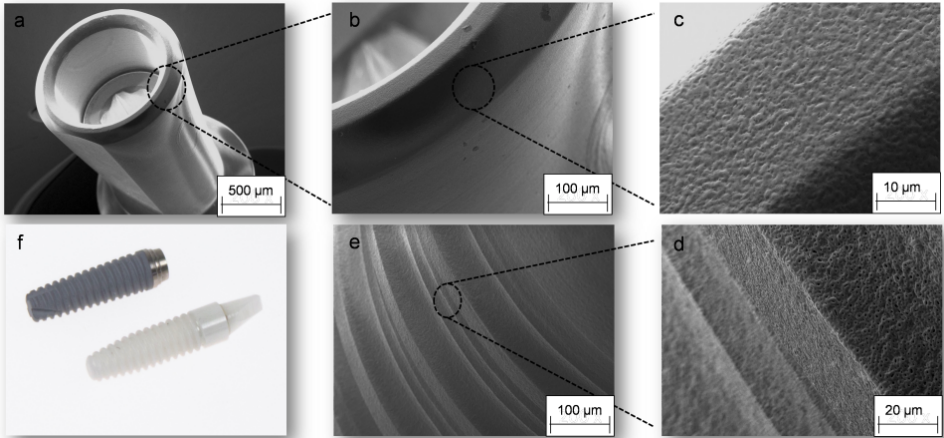
Novel ceramicized abutment and implant surfaces

The division of regenerative orofacial medicine at the UKE is also analyzing innovative surfaces to optimize the hard and soft tissue interface of dental endosseous implants. These surface modifications can, for example, increase the abrasion resistance of the implant and thus significantly reduce the risk of mucositis or peri-implantitis by favoring osseointegration and reducing abrasion. Requirements for the implant surface include good biocompatibility and biofunctionality, an increase in longevity, and high mechanical strength [15], [16]. The subject of current research is a modification of implant surfaces by means of plasma anodization (“ceramization”) to generate a more hydrophilic and porous surface and to create a more “tooth-colored/whitish” implant (Figure 13 (Fig. 13)). This is primarily intended to enhance the esthetic outcome for each patient in combination with optimized hard and soft tissue management.

**Figure 14:**
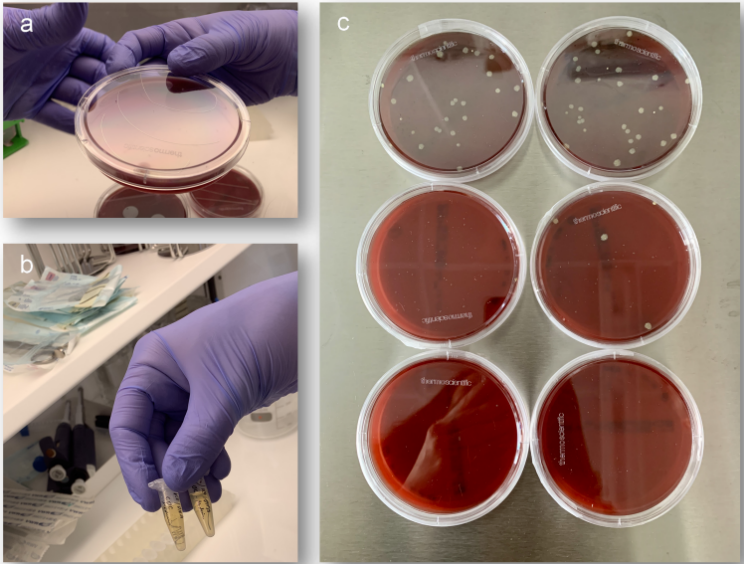
In vitro testing of an absorbable antibacterial-loaded magnesium PA filament

**Figure 15:**
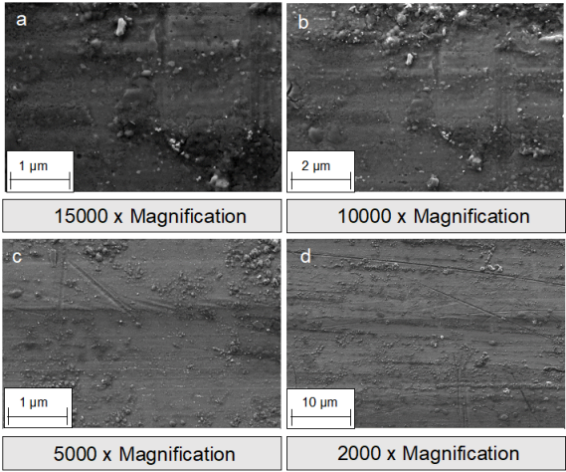
SEM images of an antibacterial-loaded Mg-PA filament

Still, the range of innovative ideas in the implantological field is far from exhausted. Antibacterial functionalization of dental implants in the crestal region is intended to prevent infections and thus the threat of peri-implant mucositis or peri-implantitis, with a current prevalence of 43 percent for peri-implant mucositis and 22 percent for peri-implantitis. In this context, the antibacterial loading dose is expected to be released continuously over a prolonged period of time, thus rendering infection-prone primary osseointegration free of inflammation. We are also currently researching an antibacterially loaded magnesium filament for periodontitis/peri-implantitis treatment (Figure 14 (Fig. 14) and Figure 15 (Fig. 15)).

### 2.5 Proprietary blood concentrates

**Figure 16:**
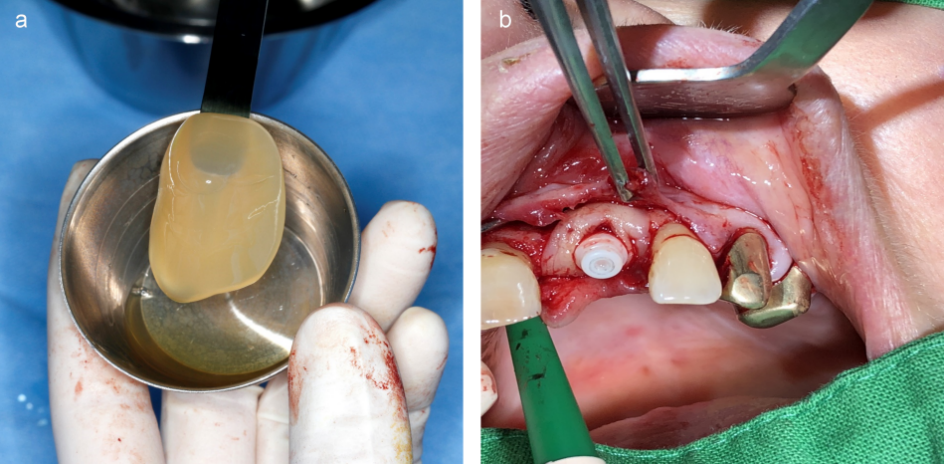
a: Autologously derived PRP membrane for use in regenerative dentistry; b: ceramic implant placement using a PRP membrane to optimize regenerative potential.

A simple idea to physiologically support healing processes in the field of dentistry is offered by autologously derived platelet-rich plasma, which is obtained by centrifugation of the patient’s own peripheral blood. The basic mechanism of action of the regenerative procedure is based on the growth factors contained in the blood plasma (including platelet-derived growth factor, transforming growth factor-β1 and 2, basic fibroblast growth factor, insulin-like growth factor, vascular endothelial growth factor and epithelial cell growth factor) [17]. Through these, endogenous healing processes can be accelerated after surgery at both a cellular and a subcellular level. At the same time, biologization and support of biomaterial integration are also possible. In addition to pre-implant and peri-implant hard tissue augmentation, autonomous PRP is also used in the treatment of periodontitis and peri-implantitis. Current studies are examining the use of PRP for the regenerative treatment of temporomandibular joint arthritis as well as for soft tissue management in implantological procedures (Figure 16 (Fig. 16)). The spectrum of possible applications for PRP in dentistry is broad, but high-quality studies are still needed, for instance, to optimize manufacturing parameters of autologous blood products for specific indications in the future. 

**Figure 17:**
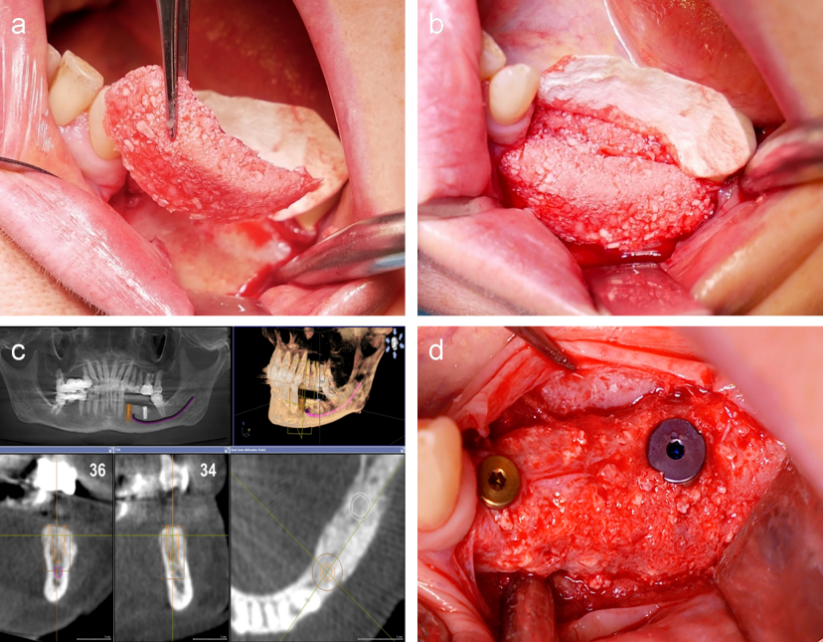
Blood plasma-stabilized augmentate (“Kieler Sushi”) for UK augmentation

In this context, our research activity in the field of blood plasma-stabilized augmentations (“Kiel Sushi”) should also be mentioned. More extensive osseous reconstructions of the alveolar ridge by guided bone regeneration (GBR) usually require complex mechanical measures to stabilize the augmentation. The “Kiel Sushi” concept solves this problem biologically through the targeted use of blood plasma. A specifically developed combination of platelets, fibrin, bone substitute material and autonomous bone not only ensures sufficient volume stability (even with large augmentations), but also enables new surgical perspectives due to its flexibility (Figure 17 (Fig. 17)).

### 2.6 Supplementation of micronutrients and vitamins

The understanding of the importance of an adequate supply of vitamins and minerals to optimally utilize the regenerative potentials of the human organism is reaching the field of dentistry and, in particular, the field of dental implantology.

Vitamins, minerals, trace elements, and their metabolites are involved in the healing process of soft tissue and bone directly or as cofactors of enzymes involved in wound healing processes. The effect and function of micronutrients and minerals on bone metabolism are undisputed. Vitamin D plays a key role in the regulation of calcium in the blood and, thus, has a direct influence on bone remodeling. Vitamin D inhibits osteoclasts and thus the resorption of bone. In the same way, it increases bone density by incorporating calcium into the bone. Vitamin D3 also decreases the expression of toll-like receptors 2 and 4 on macrophages and thereby inhibits the release of inflammatory cytokines (e.g., TNF-α). Thus, vitamin D has an anti-inflammatory effect on the human organism [18]. In the field of dentistry, it is of special interest that vitamin D inhibits the growth and virulence factors of *Porphyromonas gingivalis* [19]. Likewise, vitamin D increases the antibacterial activity of gingival epithelial cells against *Aggregatibacter actinomycetemcomitans* [20].

The association of a decreased vitamin level with periodontitis has been adequately documented in literature [21]. Vitamin D deficiency increases the risk of wound infections and may delay wound healing [22]. The correlation between early implant loss and low vitamin D levels has also been demonstrated [23].

Vitamin C also appears to play an important role, as it is necessary for the formation and cross-linking of collagen and thus for the formation of wound tissue. With the understanding of the key role in bone metabolism of vitamin D and the anti-inflammatory potential of vitamin C, it seems undeniable that an adequate supply can have a positive influence on tissue regeneration as well as on the healing of implants [24], [25], [26]. 

Thus, a deficit of vitamin D undoubtedly has a negative effect on the regenerative capacity of the bone. However, ultraviolet-induced synthesis is usually not sufficient for physiological vitamin D levels in our latitudes. The limit for an insufficient vitamin D supply is usually considered to be reached at 25(OH)D serum levels <30 ng/ml, which seems to be far off from physiological bone regeneration. An unrestricted regeneration potential with regard to vitamin D levels could be expected more readily at values between 50–70 ng/ml.

From an osteo-physiological point of view, serum vitamin D levels should therefore be continuously maintained at a value between 50–70 ng/ml in order to make optimal use of the body’s regenerative potential. Yet, there is an almost widespread vitamin D deficit in Europe, which undoubtedly has a negative effect on the regenerative capacity of bones. 

The additional substitution of vitamin K2 in a quantity ratio of 10,000 IU to 100 µg vitamin K2 can also be useful since both vitamins have a synergistic effect: vitamin K2 prevents the reversal of the otherwise positive effect of vitamin D by promoting an even deposition of calcium in the bones as well as the degradation of calcium plaques from the walls of blood vessels in case of a high vitamin D substitution (daily dose higher than 2,000 IU). 

Therefore, pre-implant nutrient and vitamin diagnostics with subsequent individual supplementation, especially in patients with reduced bone metabolism disorders (i.e., bisphosphonate intake, patients undergoing radiotherapy, osteoporosis), should be considered in the future as an option for reducing postoperative complications in implantology. 

## 3 Summary

Numerous novel materials are currently being investigated or, in some cases, are already in use to optimize the regenerative potential of hard and soft tissue in the field of dental implantology. The main focus lies particularly on absorbable material concepts for hard and soft tissue regeneration. Requirements for these materials include absorbability, biocompatibility and good integration into the surrounding tissue.

In addition to the application of innovative biomaterials, pre-implant nutrient and vitamin diagnostics can also be of great importance in order to protect patients with a reduced or impaired bone metabolism, particularly from postoperative complications. A continuous exchange of expert knowledge between scientists and practicing implantologists is essential in order to keep the requirements for novel materials conforming with the current state of research at all times and thus to ensure optimum treatment success for the patient.

## Notes

### Competing interests

The authors declare that they have no competing interests.
